# Complete Draft Genome Sequence of an Extended-Spectrum β-Lactamase-Producing Citrobacter freundii Strain Recovered from the Intestine of a House Sparrow (Passer domesticus) in Germany, 2017

**DOI:** 10.1128/genomeA.00599-18

**Published:** 2018-06-28

**Authors:** Valerie Osieka, Mirjam Grobbel, Silvia Schmoger, Claudia A. Szentiks, Alexandra Irrgang, Annemarie Käsbohrer, Bernd-Alois Tenhagen, Jens A. Hammerl

**Affiliations:** a Department of Biological Safety, German Federal Institute for Risk Assessment, Berlin, Germany; b Department of Wildlife Diseases, Leibniz Institute for Zoo and Wildlife Research (IZW), Berlin, Germany; c Institute for Veterinary Public Health, University of Veterinary Medicine, Vienna, Austria

## Abstract

Here, we announce the genome of an extended-spectrum β-lactamase-producing Citrobacter freundii strain isolated from the cecum of a house sparrow that was found dead in Berlin-Lichtenberg, Germany, in 2017. This isolate exhibits increased MICs for several antimicrobials and a comprehensive set of acquired resistance determinants potentially involved in horizontal gene transfer.

## GENOME ANNOUNCEMENT

Citrobacter freundii is a Gram-negative, opportunistic pathogen of the family *Enterobacteriaceae*. It is widely distributed in the environment and is also present in the intestine of wildlife, livestock, and humans. In the past, the pathogenic potential of this bacterium was considered low, but C. freundii is now recognized as an important nosocomial pathogen causing both superficial wound infections and life-threatening infections (1, 2), especially in neonates and elderly and immunocompromised persons (3). C. freundii has been shown to adapt efficiently to prevailing environmental conditions by acquiring genetic material from other bacteria. The uptake of various antimicrobial resistance determinants and the spread of multidrug-resistant (MDR) C. freundii clones, especially in health care units, are major public health issues (4). Further information on the biology and genetics will be needed to understand the evolution and dissemination of MDR C. freundii strains.

Extended-spectrum β-lactamase (ESBL)-producing *Enterobacteriaceae* were recovered from fecal and cecal samples of German wildlife by cultivation on MacConkey agar supplemented with 1 mg/liter cefotaxime (5). Representative colonies of each sample were identified by a MALDI Biotyper (Bruker, Bremen, Germany). The isolates were tested for their susceptibility to antimicrobial substances using the broth microdilution method according to CLSI guideline M07 (CLSI M07-A10) (6) and assessed according to EUCAST ECOFFs (http://www.eucast.org). Among all isolated C. freundii, strain 236-17-2 attracted attention because of a comprehensive antimicrobial resistance pattern, with high MIC(s) for ampicillin (>64 mg/liter), chloramphenicol (>128 mg/liter), ciprofloxacin (0.5 mg/liter), nalidixic acid (>128 mg/liter), sulfamethoxazole (>1024 mg/liter), tetracycline (64 mg/liter), trimethoprim (>32 mg/liter), cefepime (0.5 mg/liter), ertapenem (0.12 mg/liter), cefotaxime (32 mg/liter), cefoxitin (>64 mg/liter), and ceftazidime (32 mg/liter). Here, we announce the draft genome of this isolate, which was recovered from the intestine of a house sparrow (Passer domesticus) in Berlin-Lichtenberg (Germany) in 2017.

Genome sequencing was performed using DNA extracted with the PureLink Genomic DNA minikit (Invitrogen, Carlsbad, CA, USA). Short-read sequencing (MiSeq Reagent v3 600-cycle kit, Illumina, San Diego, USA) was conducted on a MiSeq benchtop sequencer using sequencing libraries prepared with the Nextera XT DNA sample preparation kit as previously described (6). *De novo* genome assembly was performed using the PATRIC database (https://www.patricbrc.org/) and resulted in 36 contigs with an average sequence coverage of 15 per consensus base. The resulting genome consists of 4,973,288 bp with an average G+C content of 51.51%. For initial genome annotation, the automated Prokaryotic Genome Annotation Pipeline of the NCBI database (7) was used, resulting in the identification of 4,856 genes, 4,753 coding sequences, 103 RNAs (16 rRNAs, 75 tRNAs, and 12 noncoding RNAs [ncRNAs]), and 130 pseudogenes. The antimicrobial resistance pattern of isolate 236-17-2 was partially confirmed by *in silico* detection of the acquired resistance genes for aminoglycosides (*strA* and *strB*), beta-lactams (*bla*_CMY-67_ and *bla*_TEM-1A_), phenicols (*catA1*), sulfonamides (*sul1*), tetracyclines [*tet(A)*], and trimethoprim (*dfrA1*) using ResFinder3.0 (https://cge.cbs.dtu.dk/services/ResFinder/). Further analyses will be necessary to determine the locations of the resistance determinants on potential mobile elements (i.e., transposons and plasmids) and their role in the transmission to other bacteria of the *Enterobacteriaceae*.

### Accession number(s).

The complete genome sequence of the C. freundii strain 236-17-2 genome was deposited in GenBank under accession number PQFB00000000.
